# Supplementary material for: Transforming Growth Factor Beta 2 (TGFB2) and Interferon Gamma Receptor 2 (IFNGR2) mRNA Levels in the Brainstem Tumor Microenvironment (TME) Significantly Impact Overall Survival in Pediatric DMG Patients
Source: Biomedicines. 2024 Jan 15;12(1):191. doi: 10.3390/biomedicines12010191 (PMC10813255; doi:10.3390/biomedicines12010191)
Supplement: Supplementary file 1 [file biomedicines-12-00191-s001.zip › biomedicines-2764446-supplementary.pdf]

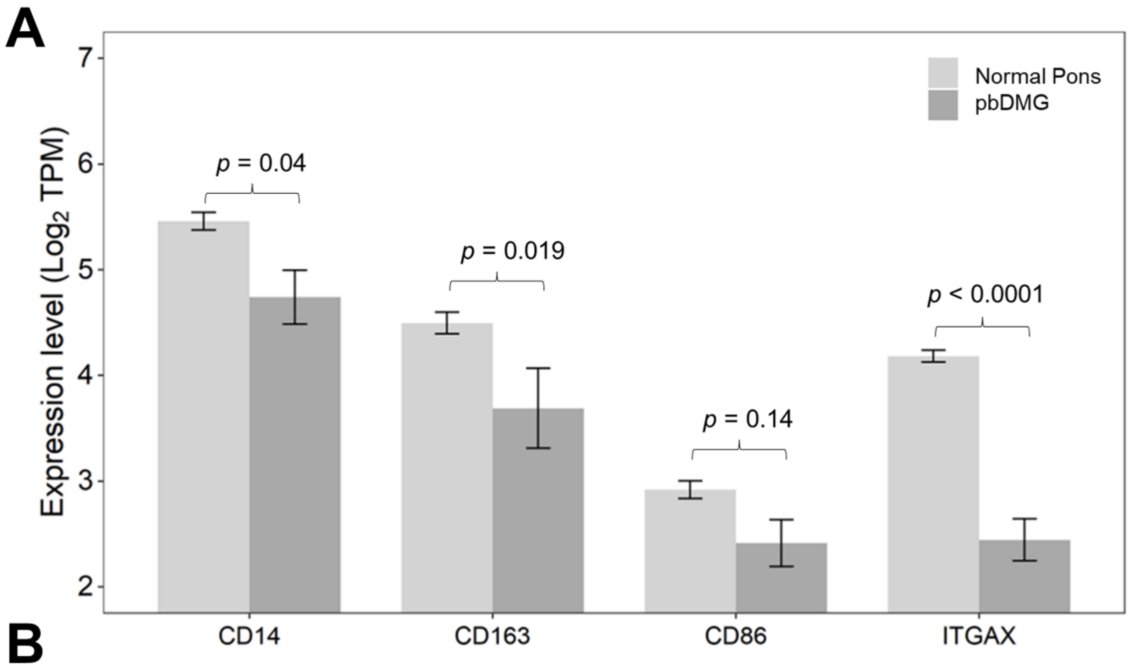

B

Gene	Normal Pons		pbDMG		Fold Change	Linear Contrasts
	Mean ± SEM	Median (Range)	Mean ± SEM	Median (Range)	pbDMG/Normal Pons	*p*-value
CD14	5.46 ± 0.08	5.38 (4.82 – 6.44)	4.74 ± 0.25	4.49 (1.8 – 9.13)	0.61	0.037
CD163	4.49 ± 0.1	4.41 (3.62 – 5.51)	3.69 ± 0.38	3.87 (−2.56 – 8.67)	0.57	0.019
CD86	2.92 ± 0.08	2.83 (2.23 – 3.92)	2.41 ± 0.22	2.46 (−1.12 – 5)	0.70	0.141
ITGAX	4.18 ± 0.06	4.19 (3.56 – 4.74)	2.44 ± 0.2	2.49 (−1.36 – 4.96)	0.30	<0.0001

Figure S1. Down-regulation of antigen-presenting cell mRNA expression in brainstem/pons of pbDMG tumors compared to normal brainstem/pons tissue.

We compared CD14 (*n*=45), CD163(*n*=45), CD86 (*n*=45), and ITGAX (*n*=45) mRNA expression levels (log2 TPM) in pbDMG samples to the expression in normal pons samples from 29 pons regions (downloaded from https://www.proteinatlas.org/about/download accessed 17th December 2022). [**A**] The bar charts illustrate mean expression levels for mRNA in tumor specimens from pbDMG patients (dark grey bars) compared to normal pons samples (light grey bars). [**B**] The statistical significance of differences in mRNA expression levels (in log2-transformed TPM values) was assessed using a two-way ANOVA with linear contrasts with gene level blocked design using FDR-adjusted *p*-values. Compared to normal brainstem/pons tissue, CD14, CD163, and ITGAX mRNA expression in pbDMG patients had significant decreases of 1.64-fold (p=0.037), 1.75-fold (p=0.019), and 3.33-fold (p<0.0001), respectively. CD86 mRNA expression in DMG patients showed a non-significant 1.42 decrease compared to normal brainstem/pons tissue (p=0.14).

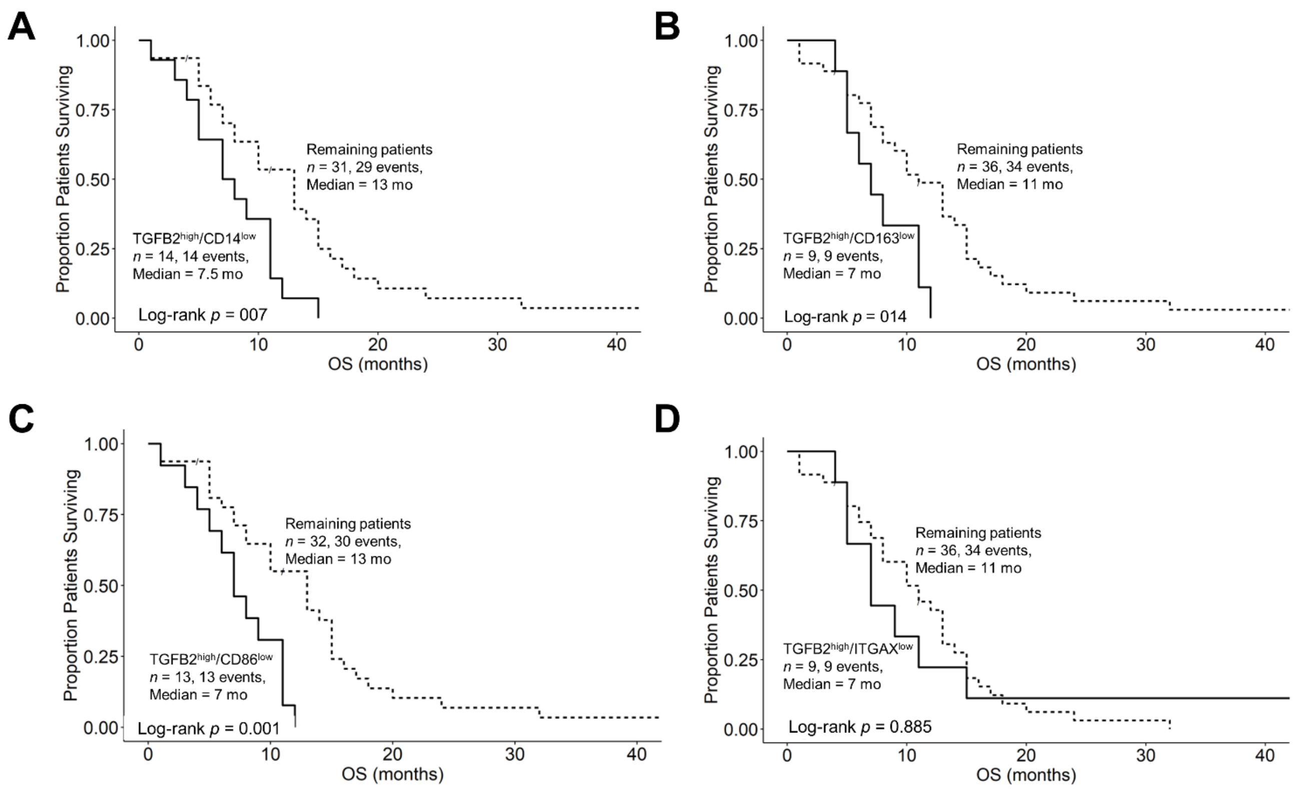

Figure S2. pbDMG patients with high levels of TGFB2 and low levels of antigen-presenting cell surface receptor mRNA expression exhibited significantly shorter OS times than the remaining patients.

We downloaded clinical metadata and RNA sequencing-based mRNA expression data for 45 patients diagnosed with pbDMG (https://pedcbioportal.kidsfirstdrc.org/study/summary?id=openpbta%2Cpbta_all). The RSEM-determined TPM metric was used to calculate the percentiles of TGFB2 and expression of CD14, CD163, CD86, and ITGAX mRNA in 45 pbDMG patients. Two patient groups were then formed based on their expression levels of TGFB2 and CD14 (**A**), CD163 (**B**), CD86 (**C**), and ITGAX (**D**), comparing higher than or equal to the 50th percentile of both TGFB2 and lower than 50th percentile for the cell surface markers with remaining patients (*n*=32). OS curves were then compared between these groups to assess the survival impact of the combination of TGFB2 and cell surface receptor levels. [**A**] The median overall survival time for 14 patients in the TGFB2^{high}/CD14^{low} group was 7.5 months (95% CI: 5 –12, number of events=14) was significantly shorter (Log-rank Chi-square=7.4, *p*-value=0.007) compared to the median survival time for 31 remaining patients of 13 months (95% CI: 8–15, number of events=29). [**B**] The median overall survival time for 9 patients in the TGFB2^{high}/CD163^{low} group was 7 months (95% CI: 5 – NA, number of events = 9) as compared to the median overall survival time for 36 remaining patients of 11 months (95% CI: 8 – 15, number of events = 34). The difference in survival outcome for these groups was statistically significant (Log-rank Chi-square = 6.06, *p*-value = 0.014). [**C**] The median overall survival time for 13 patients in the TGFB2^{high}/CD86^{low} was 7 months (95% CI: 5–NA, number of events=13) which was significantly (Log-rank Chi-square=10.5, *p*-value=0.001) shorter than the overall survival time for 32 remaining of 13 months (95% CI: 8–15, number of events=30). [**D**] The median overall survival time for 9 patients in the TGFB2^{high}/ITGAX^{low} group was 7 months (95% CI: 5−NA, number of events = 9), which was shorter but not statistically significant (*p*=0.885) compared to the median overall survival time for the remaining 36 patients of 11 months (95% CI: 8–14, number of events = 34).

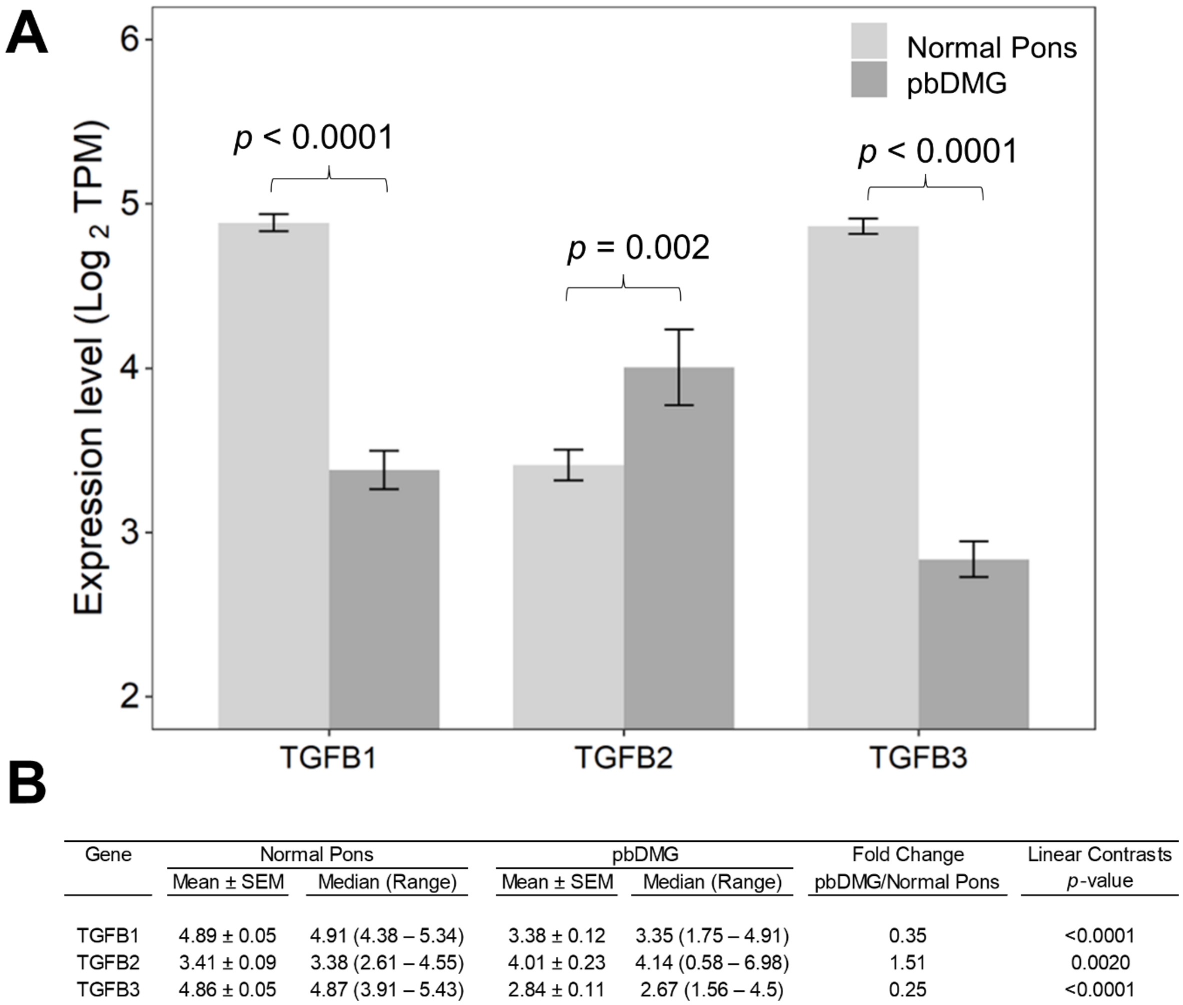

B

Gene	Normal Pons		pbDMG		Fold Change	Linear Contrasts
	Mean ± SEM	Median (Range)	Mean ± SEM	Median (Range)	pbDMG/Normal Pons	*p*-value
TGFB1	4.89 ± 0.05	4.91 (4.38 – 5.34)	3.38 ± 0.12	3.35 (1.75 – 4.91)	0.35	<0.0001
TGFB2	3.41 ± 0.09	3.38 (2.61 – 4.55)	4.01 ± 0.23	4.14 (0.58 – 6.98)	1.51	0.0020
TGFB3	4.86 ± 0.05	4.87 (3.91 – 5.43)	2.84 ± 0.11	2.67 (1.56 – 4.5)	0.25	<0.0001

Figure S3. Selective upregulation of TGFB2 mRNA expression in pbDMG patients compared to TGFB1 and TGFB3 expression levels.

TGFB1 (*n*=39; mRNA expression values were not recorded for 6 patients), TGFB2(*n*=45), TGFB3 (*n*=39; mRNA expression values were not recorded for 6 patients) mRNA expression levels (log2 TPM) in DMG samples were compared the expression in normal pons samples from 29 pons regions (downloaded from https://www.proteinatlas.org/about/download accessed 17th December 2022). [**A**] The bar charts illustrate mean expression levels for mRNA in tumor specimens from DMG patients (dark grey bars) compared to normal pons samples (light grey bars). [**B**] The statistical significance of differences in mRNA expression levels (in log2-transformed TPM values) was assessed using a two-way ANOVA with linear contrasts using FDR-adjusted *p*-values. A statistical comparison between TGFB1, TGFB2, and TGFB3 expression in DMG compared to normal pons tissue revealed a significant ($p<0.0001$, 0.002, and <0.0001 respectively) 2.84-fold decrease, 1.51-fold increase and 4.08-fold decrease in TGFB1, TGFB2 and TGFB3 mRNA expression relative to normal pons tissue respectively, suggesting that DMG brainstem tissues selectively upregulate TGFB2 and downregulate TGFB1 and TGFB3. A comparison of TGFB1 and TGFB2 mRNA in normal pons tissue revealed a significant ($p<0.0001$) 2.78-fold lower expression of TGFB2. This was also observed in the comparison of TGFB2 and TGFB3 mRNA expression that showed a highly significant ($p<0.0001$) 2.74-fold decrease in mRNA expression for TGFB2. In contrast, in DMG samples, TGFB2 mRNA expression was significantly higher than TGFB1 (1.54-fold increase; $p= 4.1\times10^{-4}$) and TGFB3 (2.25-fold increase; $p<0.0001$), suggesting specific upregulation of the TGFB2 isoform in tumor tissue.

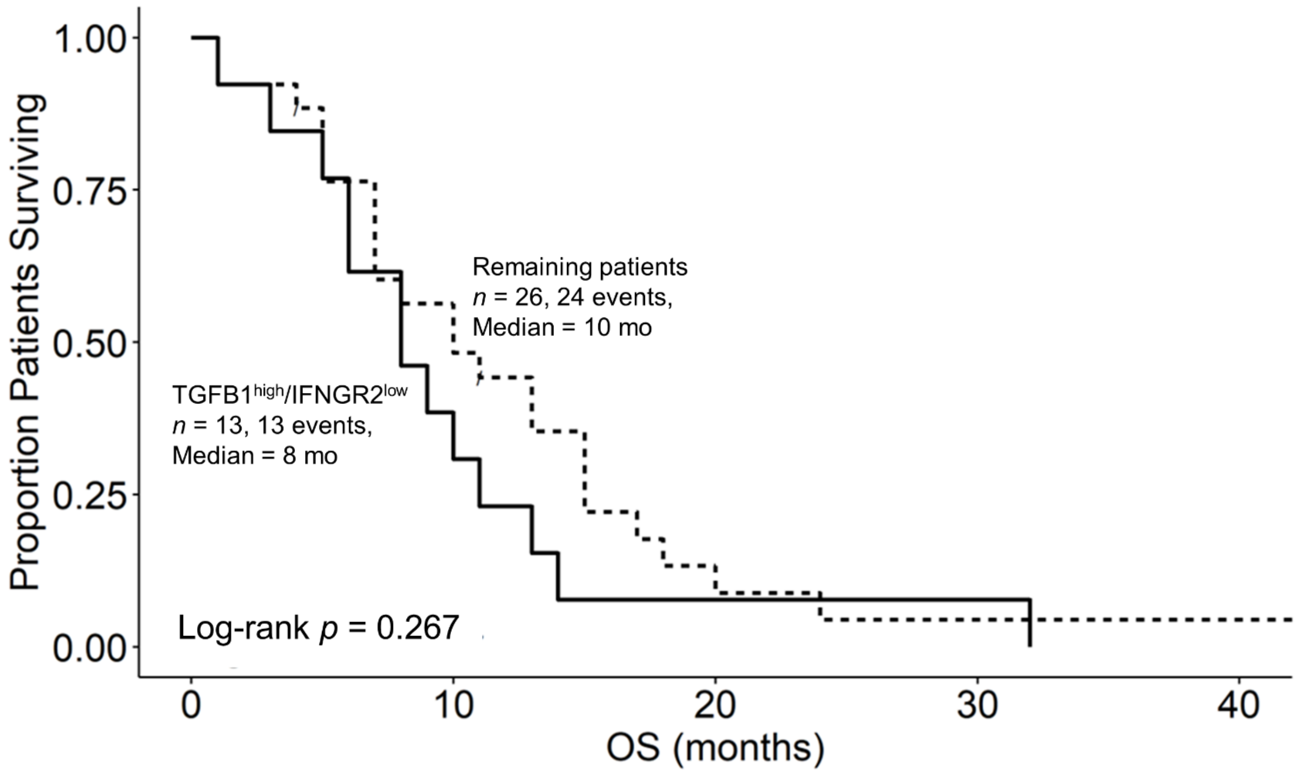

Figure S4. pbDMG patients with high levels of TGFB1 and low levels of IFNGR2 mRNA expression impact OS times compared to remaining patients.

We downloaded clinical metadata and RNA sequencing-based mRNA expression data for 39 evaluable patients diagnosed with pbDMG (https://pedcbioportal.kidsfirstdrc.org/study/summary?id=openpbta%2Cpbta_all). The RSEM-determined TPM metric was used to calculate the percentiles of TGFB1 and IFNGR2 expression in 39 evaluable pbDMG patients (mRNA expression values were not recorded for 6 patients). Two patient groups were then formed based on their expression levels of TGFB1 and IFNGR2: high expression of TGFB1 mRNA and low expression of IFNGR2 (TGFB1^{high}/IFNGR2^{low}; higher than or equal to the 50th percentile of both TGFB1 and lower than 50th percentile for IFNGR2 (*n*=13)); and remaining patients (*n*=26). OS curves were then compared between these groups to assess the survival impact of the combination of TGFB1 and IFNGR2 levels. The median OS time for 13 patients with TGFB1^{high}/IFNGR2^{low} was 8 (95% CI: 6 – NA, number of events = 13) months, while that of 26 remaining patients was 10 (95% CI: 7 – 15, number of events = 24) months. The difference in survival outcome between these two groups was not statistically significant (Log-rank Chi-Square = 1.23, *p*-value = 0.267).

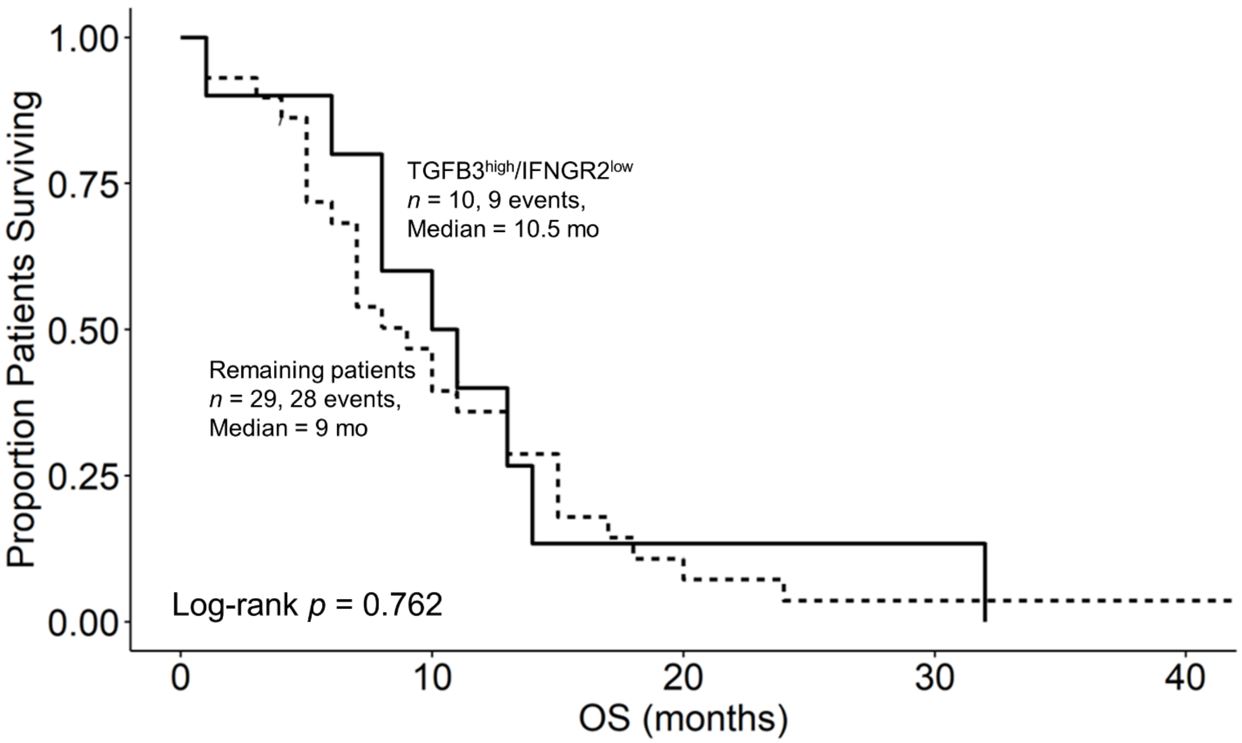

Figure S5. pbDMG patients with high levels of TGFB3 and low levels of IFNGR2 mRNA expression had no impact on OS times compared to the remaining patients.

We downloaded clinical metadata and RNA sequencing-based mRNA expression data for 39 patients diagnosed with pbDMG (https://pedcbioportal.kidsfirstdrc.org/study/summary?id=openpbta%2Cpbta_all). The RSEM-determined TPM metric was used to calculate the percentiles of TGFB3 and IFNGR2 expression in 39 evaluable pbDMG patients (mRNA expression values were not recorded for 6 patients). Two patient groups were then formed based on their expression levels of TGFB3 and IFNGR2: high expression of TGFB3 mRNA and low expression of IFNGR2 ($TGFB3^{high}/IFNGR2^{low}$; higher than or equal to the 50th percentile of both TGFB3 and lower than 50th percentile for IFNGR2 (*n*=10)); and remaining patients (*n*=29). OS curves were then compared between these groups to assess the survival impact of the combination of TGFB3 and IFNGR2 levels. The median OS time for 10 patients from the $TGFB3^{high}/IFNGR2^{low}$ group was 10.5 months (95% CI: 8–NA, number of events = 9), while the median overall survival time for 29 patients in the remaining group was 9 months (95% CI: 7 – 15, number of events = 28). The difference in survival outcome between these two groups was not statistically significant (Log-rank Chi-Square = 0.091, *p*-value = 0.762).

Table S1. Clinical trials targeting pbDMG patients.

NCT Number	Title	Conditions	Interventions	Age	Phases
NCT04196413	GD2 CAR T Cells in Diffuse Intrinsic Pontine Gliomas(DIPG) & Spinal Diffuse Midline Glioma(DMG)	Glioma of Spinal Cord<br>Glioma of Brainstem	Drug: GD2 CAR T cells<br>Drug: Fludarabine<br>Drug: Cyclophosphamide	2 to 50 yr	Phase 1
NCT04099797	C7R-GD2.CAR T Cells for Patients With GD2-expressing Brain Tumors (GAIL-B)	Diffuse Intrinsic Pontine Glioma<br>High Grade Glioma<br>Embryonal Tumor<br>Ependymal Tumor	Genetic: (C7R)-GD2.CART cells	1 to 21 yr	Phase 1
NCT05298995	GD2-CAR T Cells for Pediatric Brain Tumours	Brain Tumor, Pediatric<br>Medulloblastoma, Childhood<br>Embryonal Tumor<br>High Grade Glioma<br>Diffuse Midline Glioma<br>Diffuse Intrinsic Pontine Glioma<br>Brain Tumor Adult	Biological: GD2-CART01 (iC9-GD2-CAR T-cells)	0.5 to 30 yr	Phase 1
NCT04185038	Study of B7-H3-Specific CAR T Cell Locoregional Immunotherapy for Diffuse Intrinsic Pontine Glioma/Diffuse Midline Glioma and Recurrent or Refractory Pediatric Central Nervous System Tumors	Central Nervous System Tumor<br>Diffuse Intrinsic Pontine Glioma<br>Diffuse Midline Glioma	Biological: SCRI-CARB7H3(s); B7H3-specific chimeric antigen receptor (CAR) T cel	1 to 26 yr	Phase 1

		Ependymoma<br>Medulloblastoma, Childhood<br>Germ Cell Tumor<br>Atypical Teratoid/Rhabdoid Tumor<br>Primitive Neuroectodermal Tumor<br>Choroid Plexus Carcinoma<br>Pineoblastoma, Childhood<br>Glioma			
NCT02960230	H3.3K27M Peptide Vaccine With Nivolumab for Children With Newly Diagnosed DIPG and Other Gliomas	Diffuse Intrinsic Pontine Glioma<br>Glioma<br>Diffuse Midline Glioma, H3 K27M-Mutant	Biological: K27M peptide<br>Drug: Nivolumab	3 to 21 yr	Phase 1<br>Phase 2
NCT04749641	Neoantigen Vaccine Therapy Against H3.3-K27M Diffuse Intrinsic Pontine Glioma	Diffuse Intrinsic Pontine Glioma	Biological: Histone H3.3-K27M Neoantigen Vaccine Therapy	≥ 5 yr	Phase 1
NCT04808245	A MultIceNTER Phase I Peptide VaCcine Trial for the Treatment of H3-Mutated Gliomas	Newly Diagnosed H3-mutated Glioma	Drug: Tecentriq 1200 MG in 20 ML Injection<br>Biological: H3K27M peptide vaccine	> 18 yr	Phase 1
NCT01058850	Phase I Rindopepimut After Conventional Radiation in Children w/ Diffuse Intrinsic Pontine Gliomas	Brain Cancer<br>Brain Stem Tumors<br>Pontine Tumors	Biological: Rindopepimut	3 to 18 yr	Phase 1

NCT04978727	A Pilot Study of SurVaxM in Children Progressive or Relapsed Medulloblastoma, High Grade Glioma, Ependymoma and Newly Diagnosed Diffuse Intrinsic Pontine Glioma	Medulloblastoma	Biological: SurVaxM for patients with relapsed or progressive MB, HGG or ependymoma ages ≥10 and ≤21 years	≥10 and ≤21 yr	Phase 1
		Glioblastoma Multiforme			
		Anaplastic Astrocytoma	Biological: SurVaxM for patients with relapsed or progressive MB, HGG or ependymoma ages ≥1 and <10 years	≥1 and <10 yr	
		High-grade Astrocytoma NOS			
		Anaplastic Oligodendroglioma	Biological: SurVaxM for patients with non-relapsed DIPG post radiation-therapy ages ≥1 and ≤21 years	≥1 and ≤21 yr	
		Anaplastic Ependymoma			
		Ependymoma			
		Diffuse Intrinsic Pontine Glioma			
NCT04943848	rHSC-DIPGVax Plus Checkpoint Blockade for the Treatment of Newly Diagnosed DIPG and DMG	Diffuse Intrinsic Pontine Glioma	Biological: rHSC-DIPGVax	0.5 to 18 yr	Phase 1
		Diffuse Midline Glioma, H3 K27M-Mutant	Drug: Balstilimab		
			BALSTILIMAB is a human monoclonal antibody that targets programmed cell death 1 (PD1)		
			Drug: Zalifrelimab		
			ZALIFRELIMAB is a human monoclonal immunoglobulin G1k subclass (IgG1k) antibody that specifically recognizes cytotoxic T lymphocyte-associated protein 4 (CTLA-4, also known as CD152)		
NCT03330197	A Study of Ad-RTS-hIL-12 + Veledimex in Pediatric Subjects With Brain Tumors Including DIPG	Pediatric Brain Tumor	Biological: Ad-RTS-hIL-12	≤ 21 yr	Phase 1
		Diffuse Intrinsic Pontine Glioma	Drug: Oral Veledimex - Arm 1 (Pediatric Brain Tumor)		Phase 2

			Drug: Oral Veledimex - Arm 2 (DIPG)		
NCT03178032	Oncolytic Adenovirus, DNX-2401, for Naive Diffuse Intrinsic Pontine Gliomas	Brainstem Glioma<br>Neoadjuvant Therapy	Biological: DNX-2401<br>Brain infusion of the virus through the cerebellar peduncle	1 to 18 yr	Phase 1
NCT02444546	Wild-Type Reovirus in Combination With Sargramostim in Treating Younger Patients With High-Grade Relapsed or Refractory Brain Tumors	Childhood Astrocytoma<br>Childhood Atypical Teratoid/Rhabdoid Tumor<br>Diffuse Intrinsic Pontine Glioma<br>Glioma<br>Recurrent Childhood Anaplastic Oligodendroglioma<br>Recurrent Childhood Brain Neoplasm<br>Recurrent Childhood Glioblastoma<br>Recurrent Childhood Medulloblastoma<br>Recurrent Primitive Neuroectodermal Tumor<br>Refractory Brain Neoplasm	Other: Laboratory Biomarker Analysis<br>Biological: Sargramostim<br>Biological: Wild-type Reovirus	10 to 21 yr	Phase 1
NCT04758533	Clinical Trial to Assess the Safety and Efficacy of AloCELYVIR With Newly Diagnosed Diffuse Intrinsic Pontine Glioma (DIPG) in Combination With Radiotherapy or Medulloblastoma in Monotherapy	Diffuse Intrinsic Pontine Glioma<br>Medulloblastoma, Childhood, Recurrent	Biological: AloCELYVIR<br>Mesenchymal allogenic cells + ICOVIR-5: 500.000 cells/kg	1 to 21 yr	Phase 1<br>Phase 2

NCT05096481	PEP-CMV Vaccine Targeting CMV Antigen to Treat Newly Diagnosed Pediatric HGG and DIPG and Recurrent Medulloblastoma	High Grade Glioma<br>Diffuse Intrinsic Pontine Glioma<br>Recurrent Medulloblastoma	Biological: PEP-CMV<br>Drug: Temozolomide<br>Biological: Tetanus Diphtheria Vaccine	3 to 25 yr	Phase 2
NCT01952769	Anti PD1 Antibody in Diffuse Intrinsic Pontine Glioma	DIPG	Biological: MDV9300<br>1. Evaluation of MDV9300 and radiation-<br>2. Evaluation of MDV9300 and cyclophosphamide<br>Other Names:<br>pidilizumab	3 to 21 yr	Phase 1<br>Phase 2
NCT02359565	Pembrolizumab in Treating Younger Patients With Recurrent, Progressive, or Refractory High-Grade Gliomas, Diffuse Intrinsic Pontine Gliomas, Hypermutated Brain Tumors, Ependymoma or Medulloblastoma	Constitutional Mismatch Repair Deficiency Syndrome<br>Lynch Syndrome<br>Malignant Glioma<br>Recurrent Brain Neoplasm<br>Recurrent Childhood Ependymoma<br>Recurrent Diffuse Intrinsic Pontine Glioma<br>Recurrent Medulloblastoma<br>Refractory Brain Neoplasm<br>Refractory Diffuse Intrinsic Pontine Glioma<br>Refractory Ependymoma<br>Refractory Medulloblastoma	Biological: Pembrolizumab	1 to 30 yr	Phase 1

NCT03690869	REGN2810 in Pediatric Patients With Relapsed, Refractory Solid, or Central Nervous System (CNS) Tumors and Safety and Efficacy of REGN2810 in Combination With Radiotherapy in Pediatric Patients With Newly Diagnosed or Recurrent Glioma	Relapsed Solid Tumor<br>Refractory Solid Tumor<br>Relapsed Central Nervous System Tumor<br>Refractory Central Nervous System Tumor<br>Diffuse Intrinsic Pontine Glioma<br>High Grade Glioma	Drug: cemiplimab (monotherapy)<br>To be administered intravenously as monotherapy in Phase 1<br>Other Names:<br>REGN2810<br>Libtayo<br>Drug: cemiplimab (maintenance)<br>To be administered intravenously in combination with radiation and then used as maintenance therapy<br>Other Names:<br>REGN2810<br>Libtayo<br>Radiation: Conventional or hypofractionated<br>Combined with cemiplimab IV administration<br>Radiation: Re-irradiation<br>Combined with cemiplimab IV administration	0 to <18 yr (Phase 1)<br>≥3 and ≤25 yr (Efficacy Phase)	Phase 1<br>Phase 2

Table S2. Patient characteristics for pbDMG, High Grade Glioma (HGG) and Low Grade Glioma (LGG) patients

Variable	pbDMG Mean/Median(Range) or *n* (% evaluable)		HGG Mean/Median(Range) or *n* (% evaluable)		LGG Mean/Median(Range) or *n* (% evaluable)	
**Age (yrs)**						
Mean±SEM / Median (Range)	7.38 ± 0.59	7 (2 – 18)	9.26 ± 0.41	9 (0 – 25)	8.09 ± 0.28	8 (0 – 31)
n	45		171		404	
**Fraction Genome Altered**						
Mean±SEM / Median (Range)	0.22 ± 0.04	0.16 (0 – 0.81)	0.32 ± 0.02	0.26 (0 – 0.92)	0.16 ± 0.01	0.07 (0 – 0.99)
n	25		101		363	
**Mutation Count**						
Mean±SEM / Median (Range)	44 ± 19	23 (2 – 499)	485 ± 217	32 (3 – 12800)	23 ± 8	9 (1 – 2677)
n	25		101		362	
**Sex**						
Female	29(66%)		85(49%)		183(45%)	
Male	15(34%)		88(51%)		222(55%)	
**Ethnicity**						
Hispanic or Latino	5(13%)		27(19%)		43(11%)	
Not Hispanic or Latino	35(87%)		114(81%)		336(89%)	
**Race**						
All Other	9(20%)		49(28%)		81(20%)	
Asian	4(9%)		9(5%)		8(2%)	
Black or African American	4(9%)		10(6%)		27(7%)	
White	28(62%)		106(61%)		289(71%)	
